# Supplementary material for: Sonochemical Synthesis, Characterization and Optical Properties of Tb-Doped CdSe Nanoparticles: Synergistic Effect between Photocatalysis and Sonocatalysis
Source: Nanomaterials (Basel). 2021 Feb 2;11(2):378. doi: 10.3390/nano11020378 (PMC7913114; doi:10.3390/nano11020378)
Supplement: Supplementary file 1 [file nanomaterials-11-00378-s001.pdf]

Table S1. CdSe based photocatalysts and their photo or soncatalytic applications.

**Photocatalyst**	**Target**	**Experimental conditions**	**Activity**	**Ref.**
CdSe nanotube (B.G.=1.7 eV)	Methylene Blue	Photocatalysis, 150-W xenon lamp (I = 100 W/m^2), 0.5 mg/L of Pollutant.	~35% (180 min)	[1]
Dy doped CdSe (B.G.=1.7 eV)	Anazolene sodium	Sonocatalysis, ultrasonic bath (150 W) 10 mg/L of Pollutant and 1000 mg/L of Cat.	91.3% (90 min)	[2]
CdSe quantum dots (B.G.=1.53 eV)	cefalexin	Photocatalysis, 300 W high pressure Hg lamp (UV, 365 nm), 15 mg/L of Pollutant and 500 mg/L of Cat.	70.34% (120 min)	[3]
CdSe and $CdSe/TiO_2$	4-chlorophenol	Photocatalysis, 300 W tungsten halogen lamp, 2.5×10^{-4} M of Pollutant and 200 mg/L of Cat.	~12% by CdSe and 32% by $CdSe/TiO_2$ (480 min)	[4]
Zn-doped CdSe	Methylene blue and moxifloxacin	Photocatalysis, UV-A (485 μW cm^{-2} , 300–440 nm with main peak at 365 nm) lamp, 5 mg/L of Pollutant and 200 mg/L of Cat for MB, 15 mg/L of Pollutant and 500 mg/L of Cat for MOX.	93% for MB (90 min), 81.6% for MOX (110 min)	[5]
Au-CdSe Pentapod Heterostructures	Rhodamine 6G	Photocatalysis, UV irradiation (365 nm), 2.18×10^{-6} M of Pollutant.	~88% (150 min)	[6]
CdSe and CdSe/C60	Methylene Blue	Photocatalysis, halogen lamps, 8 W, 1×10^{-5} M of Pollutant and 500 mg/L of Cat.	~95% (300 min) for CdSe/C60	[7]
CdSe and CdSe/CdS core–shell QDs	Methyl orange	Photocatalysis, halogen lamps, 8 W, 10 mg/L of Pollutant and 200 mg/L of Cat.	73% by CdSe and 92% by CdSe/CdS (60 min)	[8]
Vesicle CdSe	Tetracycline hydrochloride	Photocatalysis, 500 W Xe lamp, 20 mg/L of Pollutant and 500 mg/L of Cat.	78.82% (30 min)	[9]
K and Co codoped CdSe QDs	Tetracycline hydrochloride	Photocatalysis, 500 W Xe lamp, 20 mg/L of Pollutant and 500 mg/L of Cat.	~83% (30 min)	[10]
Gd-doped CdSe	Acid Blue 5	Sonocatalysis, ultrasonic bath 300 W/L, 10 mg/L of Pollutant and 100 mg/L of Cat.	86% (90 min)	[11]
Graphene quantum dots/CdSe	Methylene Blue	Sonocatalysis, ultrasonic bath 200 W/L, 20 mg/L of Pollutant and 100 mg/L of Cat.	90% (90 min)	[12]
CdSe–graphene	Methyl orange and Rhodamine B	Sonocatalysis, Ultrasonic apparatus 750 W, 1×10^{-4} M mg/L of Pollutant and 10000 mg/L of Cat.	100% for RhB (150 min), 94% for MO (150 min)	[13]
Tb doped CdSe	RB5	Sonophotocatalysis, 40W compact fluorescent visible light lamp and sonicator output intensity of 200 W, 20 mg/L of Pollutant and 1120 mg/L of Cat.	87.53% (60 min)	This Work

Refrences:

1. Zhu, H.; Li, Q. Visible light-driven CdSe nanotube array photocatalyst. *Nanoscale Research Letters* **2013**, *8*, 230, doi:10.1186/1556-276X-8-230.
2. Khataee, A.; Mohamadi, F.T.; Rad, T.S.; Vahid, B. Heterogeneous sonocatalytic degradation of anazolene sodium by synthesized dysprosium doped CdSe nanostructures. *Ultrason. Sonochem.* **2018**, *40*, 361-372, doi:https://doi.org/10.1016/j.ultsonch.2017.07.021.
3. Liu, X.; Ma, C.; Yan, Y.; Yao, G.; Tang, Y.; Huo, P.; Shi, W.; Yan, Y. Hydrothermal Synthesis of CdSe Quantum Dots and Their Photocatalytic Activity on Degradation of Cefalexin. *Ind. Eng. Chem. Res.* **2013**, *52*, 15015-15023, doi:10.1021/ie4028395.
4. Ho, W.; Yu, J.C. Sonochemical synthesis and visible light photocatalytic behavior of CdSe and CdSe/TiO2 nanoparticles. *J. Mol. Catal. A: Chem.* **2006**, *247*, 268-274, doi:https://doi.org/10.1016/j.molcata.2005.11.057.
5. Asere, T.G.; Laing, G.D. Zn-doped CdSe nanoparticles: Impact of synthesis conditions on photocatalytic activity. *Environmental Technology & Innovation* **2020**, *20*, 101126, doi:https://doi.org/10.1016/j.eti.2020.101126.
6. Haldar, K.K.; Sinha, G.; Lahtinen, J.; Patra, A. Hybrid Colloidal Au-CdSe Pentapod Heterostructures Synthesis and Their Photocatalytic Properties. *ACS Applied Materials & Interfaces* **2012**, *4*, 6266-6272, doi:10.1021/am301859b.
7. Meng, Z.-D.; Zhu, L.; Oh, W.-C. Preparation and high visible-light-induced photocatalytic activity of CdSe and CdSe-C60 nanoparticles. *J. Ind. Eng. Chem.* **2012**, *18*, 2004-2009, doi:https://doi.org/10.1016/j.jiec.2012.05.019.
8. Abbasi, S.; Molaei, M.; Karimipour, M. CdSe and CdSe/CdS core–shell QDs: New approach for synthesis, investigating optical properties and application in pollutant degradation. *Luminescence* **2017**, *32*, 1137-1144, doi:https://doi.org/10.1002/bio.3300.
9. Wen, J.; Ma, C.; Huo, P.; Liu, X.; Wei, M.; Liu, Y.; Yao, X.; Ma, Z.; Yan, Y. Construction of vesicle CdSe nano-semiconductors photocatalysts with improved photocatalytic activity: Enhanced photo induced carriers separation efficiency and mechanism insight. *Journal of Environmental Sciences* **2017**, *60*, 98-107, doi:https://doi.org/10.1016/j.jes.2016.12.023.
10. Ma, C.; Zhou, M.; Wu, D.; Feng, M.; Liu, X.; Huo, P.; Shi, W.; Ma, Z.; Yan, Y. One-step hydrothermal synthesis of cobalt and potassium codoped CdSe quantum dots with high visible light photocatalytic activity. *CrystEngComm* **2015**, *17*, 1701-1709, doi:10.1039/C4CE02414A.
11. Khataee, A.; Karimi, A.; Hasanzadeh, A.; Joo, S.W. Kinetic modeling of sonocatalytic performance of Gd-doped CdSe nanoparticles for degradation of Acid Blue 5. *Ultrason. Sonochem.* **2017**, *39*, 344-353, doi:https://doi.org/10.1016/j.ultsonch.2017.04.022.
12. Sajjadi, S.; Khataee, A.; Kamali, M. Sonocatalytic degradation of methylene blue by a novel graphene quantum dots anchored CdSe nanocatalyst. *Ultrason. Sonochem.* **2017**, *39*, 676-685, doi:https://doi.org/10.1016/j.ultsonch.2017.05.030.
13. Ghosh, T.; Ullah, K.; Nikam, V.; Park, C.-Y.; Meng, Z.-D.; Oh, W.-C. The characteristic study and sonocatalytic performance of CdSe–graphene as catalyst in the degradation of azo dyes in aqueous solution under dark conditions. *Ultrason. Sonochem.* **2013**, *20*, 768-776, doi:https://doi.org/10.1016/j.ultsonch.2012.09.005.
